# Editorial: Proceedings of the 3rd International Conference on Genetics of Aging and Longevity

**DOI:** 10.3389/fgene.2016.00119

**Published:** 2016-06-24

**Authors:** Elena G. Pasyukova, Alexey Moskalev

**Affiliations:** ^1^Laboratory of Genome Variation, Institute of Molecular Genetics, Russian Academy of SciencesMoscow, Russia; ^2^Radiation Ecology, Laboratory of Molecular Radiobiology and Gerontology, Institute of Biology of Komi Science Center of Ural Branch of RASSyktyvkar, Russia; ^3^Syktyvkar State UniversitySyktyvkar, Russia; ^4^Moscow Institute of Physics and TechnologyDolgoprudny, Russia

**Keywords:** redox, telomere shortening, geroprotectors, nervous system, polymorphisms

In April 2014, the 3rd International Conference ≪Genetics of aging and longevity≫ took place in Sochi, Russia. We are pleased to present a collection of articles based on or related to the topics of the Conference and published in Frontiers in Genetics of Aging in 2015. They reflect understanding complex interactions between genes and genetic pathways underlying aging that is of utmost significance for the future life of humanity. Accordingly, genetic basis of longevity remained the most important topic of the Conference. The role of genes involved in energy supply and mitochondrial integrity and function deserved a close attention in two reviews published in this collection (Morrow and Tanguay; Rogers and Rogina). The importance of redox homeostasis in aging was considered in the paper by Klichko et al. Undoubtedly, energy metabolism governed by various genes and environmental factors, such as calorie intake, represents one of the key players in longevity control. Klichko et al. also demonstrated that temporal regulation of redox status is related to the aging-associated loss of a proper circadian modulation with age. Having joined an interesting extramural discussion, Koliada et al. reviewed the current views on the telomere length as a marker and/or a cause of aging and came to the conclusion that telomere may not serve as a “clock” counting cell divisions, but that telomere shortening, rather, reflects the history of exposure to oxidative stress in cell lineages. Thereto, another article described age-associated alterations in the chromosome morphology in a particular type of cells and indicated molecular mechanisms underlying these changes (Lebedeva et al.).

Aging is defined as a gradual loss of physiological functions accompanied by decreasing fertility and increasing risk of mortality. An important and challenging question for the world-wide scientific community is whether there are means allowing to combat aging and to prolong health span and life span. Therefore, it is not surprising that anti-aging interventions attracted much attention at the Conference and, therefore, a number of articles in this collection were concentrated on possible pharmacological agents aimed to slow down aging (Carretero et al.; Johnson et al.; Semenkov et al.; Sycheva). Of note, effects of treatments on age-related traits, such as weight, as well as on some pathological states, rather than on aging and longevity were often considered (Johnson et al.; Semenkov et al.; Sycheva). In two research papers (Johnson et al.; Semenkov et al.), anti-aging interventions were to apply chemical (rapamycin) and physical (temperature, UV light) agents targeted at TOR signaling and ROS production; the role of vitamins in reducing cellular damage was regarded by Sycheva; five main pharmacological classes of compounds known to extend lifespan were considered in the review written by Carretero et al.). Arguments supporting the idea that the nervous system is an important target for anti-aging prophylaxis were also discussed by Omelyanchuk et al. The wide spectrum of agents and targets considered in a restricted number of articles once again sends us a trivial, albeit important message: modulation and fine tuning of multiple molecular mechanisms are needed to ensure longevity. Likewise, analysis of genome-wide screens for polymorphisms significantly associated with human longevity (Yashin et al.) brought authors to the conclusion that genes involved in the life span control affect various molecular functions but, eventually, these functions converge in the development of aging and key age-related pathologies. In the context of the search for means that are able to prolong the life span, Baranova and Willett suggested to pay a close attention to “the world of metabolites,” arguing that metabolome might be the most effective target for anti-aging interventions.

Overall, articles from this collection reflect a wide range of current aging research and highlight its prospects and future directions. We hope that they will attract the attention of a broad scientific readership.

## Author contributions

All authors listed, have made substantial, direct and intellectual contribution to the work, and approved it for publication.

### Conflict of interest statement

The authors declare that the research was conducted in the absence of any commercial or financial relationships that could be construed as a potential conflict of interest.

